# The Jembrana disease virus Rev protein: Identification of nuclear and novel lentiviral nucleolar localization and nuclear export signals

**DOI:** 10.1371/journal.pone.0221505

**Published:** 2019-08-22

**Authors:** Claude Marchand, Guy Lemay, Denis Archambault

**Affiliations:** 1 Département des Sciences Biologiques, Université du Québec à Montréal, Montréal, Québec, Canada; 2 Département de Microbiologie, Infectiologie et Immunologie, Université de Montréal, Montréal, Québec, Canada; 3 Centre d’Excellence en Recherche sur les Maladies Orphelines - Fondation Courtois (CERMO-FC), Université du Québec à Montréal, Montréal, Québec, Canada; University of Padua, ITALY

## Abstract

The lentiviral Rev protein, which is a regulatory protein essential for virus replication, has been first studied in the human immunodeficiency virus type 1 (HIV-1). The main function of Rev is to mediate the nuclear exportation of viral RNAs. To fulfill its function, Rev shuttles between the cytoplasm and the nucleus. The Jembrana disease virus (JDV), a lentivirus, is the etiologic agent of the Jembrana disease which was first described in Bali cattle in Indonesia in 1964. Despite the high mortality rate associated with JDV, this virus remains poorly studied. Herein the subcellular distribution of JDV Rev, the nuclear and nucleolar localization signals (NLS and NoLS, respectively) and the nuclear export signal (NES) of the protein were examined. JDV Rev fused to the enhanced green fluorescent protein (EGFP) predominantly localized to the cytoplasm and nucleolus of transfected cells, as determined by fluorescence microscopy analyses. Through transfection of a series of deletion mutants of JDV Rev, it was possible to localize the NLS/NoLS region between amino acids (aa) 74 to 105. By substituting basic residues with alanine within this sequence, we demonstrated that the JDV Rev NLS encompasses aa 76 to 86, and is exclusively composed of arginine residues, whereas a bipartite NoLS was observed for the first time in any retroviral Rev/Rev-like proteins. Finally, a NES was identified downstream of the NLS/NoLS and encompasses aa 116 to 128 of the JDV Rev protein. The JDV Rev NES was found to be of the protein kinase A inhibitor (PKI) class instead of the HIV-1 Rev class. It also corresponds to the most optimal consensus sequence of PKI NES and, as such, is novel among lentiviral Rev NES.

## Introduction

Lentiviruses constitute a distinct viral genus within the *Retroviridae* family which includes human (HIV), simian (SIV), feline (FIV) and bovine (BIV) immunodeficiency viruses, equine infectious anemia virus (EIAV), caprine arthritis-encephalitis virus (CAEV) and Maedi-Visna virus (MVV) in sheep [1, 2]. These viruses share a relatively long incubation period followed by a protracted symptomatic phase even though the host responds immunologically. Chronic and degenerative pathologic changes also characterize lentiviral infections [3].

The Jembrana disease virus (JDV), a bovine lentivirus, is the etiologic agent of the Jembrana disease which was first described in cattle on the Island of Bali in Indonesia in 1964. In contrast to BIV, which is associated with a chronic and mostly asymptomatic infection in its natural host [4, 5], JDV leads to death in 15–17% of Bali cattle within 5 to 8 weeks post infection whereas the other animals recover and remain asymptomatic [6]. Consequently, in contrast to the chronic infection state associated with other lentiviruses like HIV type 1 (HIV-1) and BIV, JDV is unique in the sense that it may be considered as an acute infection-causing lentivirus. Despite its peculiar pathogenicity compared to other lentiviruses, JDV has been poorly studied, especially at the molecular level. This might be due to the fact that *in vitro* cell cultures have not been identified so far to allow JDV replication [7]. Consequently, data available for JDV have been obtained from raw tissue materials of animals infected with the virus [6, 8, 9].

JDV has the shortest genome (7.732 kb in length) of any lentiviruses [6]. JDV provirus DNA has a typical retroviral genome structure containing the *gag*, *pol* and *env* genes with the presence of long terminal repeats (LTRs) at the 5' and 3' termini. It also contains several regulatory/accessory genes that encode proteins, some of which are involved in the regulation of virus gene expression. Among the latter is the Rev (Regulator of viral expression) protein. JDV Rev is a 23-kDa [213 amino acids (aa)] phosphoprotein produced from a multiply spliced mRNA that contains two encoding exons located in the *env* gene [10]. The main function of lentiviral Rev protein is to mediate the nuclear exportation of partially spliced viral RNAs encoding structural proteins, and of unspliced viral RNAs that serve as genomic RNA. Rev exerts its function by interacting with a stem-loop structure termed Rev responsive element (RRE) located within these RNAs [11]. Lentiviral Rev protein contains at least three functional domains: i) a basic arginine-rich domain that mediates RNA binding (RBD) and contains the nuclear/nucleolar localization signals (NLS/NoLS) necessary for the protein transport from the cytoplasm to the nucleus and nucleolus; ii) a multimerization domain; and iii) a leucine-rich domain that contains a nuclear export signal (NES) necessary for Rev exportation from the nucleus to the cytoplasm [12].

The nuclear import of lentiviral Rev proteins into the cell nucleus is mediated by the direct binding of their NLS to nuclear transport receptors such as importin β, transportin, importins 5 and 7, and importins α/β described in HIV-1 and BIV Rev, respectively [13, 14]. Two types of classical NLSs recognized by importin α have been reported: monopartite and bipartite. The monopartite NLSs, which are composed of a single cluster of basic aa, are currently categorized into five classes on the basis of aa composition and of their interaction with importins [15]. Bipartite NLSs are composed of two clusters of basic aa that are separated by a spacer region. They are classified into two types according to the length of the spacer region: the long type with a spacer of 30 to 32 aa, and the short type with a spacer of 8 to 16 aa [16]. The NLSs of HIV-1 and EIAV Rev are composed of a single cluster of basic residues but only the NLS of EIAV Rev has been proven to be monopartite [17, 18], whereas that of BIV Rev is the only lentivirus Rev characterized so far that has a bipartite structure [19]. In addition to NLSs, lentiviral Rev proteins contain a NoLS that mediates their accumulation in the cell nucleoli [17, 19]. These NoLSs correspond to one or multiple copies of the R/K-R/K-X-R/K consensus motif [20] and have been reported in lentiviral Rev proteins to be either intrinsically associated with the NLS, as described in HIV-1 [17], or present in the spacer sequence of a bipartite NLS, as reported in BIV [19].

To date, there has been no report on the localization of JDV Rev in cells and on the functional domains of the protein. In the present study, the subcellular distribution of JDV Rev fused to enhanced green fluorescent protein (EGFP) was thus examined and the NLS, NoLS and NES of the protein were identified. The JDV Rev protein was shown to predominantly localize to the cytoplasm and nucleolus. The JDV Rev was also shown to be exported from the nucleus via a CRM1 (Chromosomal Maintenance 1) protein pathway like HIV-1 and BIV Rev. By generating a series of deletion and site-specific mutants, a JDV Rev monopartite-like NLS was mapped. Moreover, and in contrast to other lentiviral Rev NoLS, the JDV Rev NoLS was found to be bipartite in structure. Finally, the NES of JDV Rev was found to be of the protein kinase A inhibitor (PKI) class like the BIV Rev NES. However, the NES of JDV Rev, in contrast to that of BIV Rev, corresponds to the most optimal consensus sequence of PKI NES.

## Materials and methods

### Cell cultures and transfection

All cells used in this study were exempt of *Mycoplasma*, as determined by using the e-Myco VALiD Mycoplasma PCR detection kit (iNtRON Biotechnology, Burlington, MA). Bovine macrophages (BoMac) [21], Madin-Darby Bovine Kidney (MDBK) epithelial cells (ATCC: CCL-22) [22] and HEK293T cells (ATCC: CRL-3216) were maintained at 37°C in a humidified atmosphere of 5% CO_2_ in Dulbecco’s modified Eagle’s medium (DMEM for HEK293T), Eagles’s minimum essential medium (EMEM for MDBK cells) or Roswell Park Memorial Institute (RPMI) 1640 medium (for BoMac cells) supplemented with 10% fetal bovine serum (PAA Laboratories Inc., Etobicoke, Ontario, Canada). For transfections, the cells were plated at a cell density of ~ 50% confluence in 24-well cell culture plates (used for the microscopy analyses) or in 6-well cell culture plates (used for the Rev activity assay). The next day, plasmids were mixed with Xtreme Gene 9 transfection reagent (Roche, Indianapolis, IN) and added to the cells according to the manufacturer’s protocol.

### Plasmid constructs encoding the JDV Rev WT and deletion mutant proteins

The gene encoding the JDV Rev WT protein of the Tabanan/87 strain (GenBank accession number U21603.1) was kindly provided by Dr Moira Desport (Murdoch University, Perth, Australia) [6]. The gene was cloned into the pEGFP-C1 expression vector (Clontech, Palo Alto, CA) to generate a plasmid construct (pEGFP-JDV Rev WT) able to produce EGFP fused to the JDV Rev WT protein (EGFP-JDV Rev WT). Ten JDV Rev mutant (JM) proteins containing internal deletions of 32 (for JM1) or 20 (for JM2 to JM10) aa were generated from the pEGFP-JDV Rev WT construct by PCR-ligation-PCR mutagenesis [23]. Briefly, upstream and downstream blunt-ended cDNA fragments were amplified from the pEGFP-JDV Rev WT plasmid construct with appropriate phosphorylated primers (sequences available upon request). Fragments were purified and ligated to the corresponding upstream fragment. Ligation products were amplified by using the forward Rev5’ (5’- TCCGAATTCTATGATGGAAGAAGG -3’) and reverse Rev3’ (5’- GCAAGGGCCCACTGGGCG-TATTCC -3’) primers that introduced EcoRI and ApaI restriction sites (underlined nucleic acids), respectively. The generated PCR products were then digested with EcoR1 and ApaI and cloned back into the pEGFP-C1 expression vector. The N-terminal (JM1) and C-terminal (JM10) mutant sequences were amplified using a forward primer containing an ATG initiation codon and a reverse primer containing a stop codon, respectively.

To generate the pEGFP-NLS JDV construct, the nucleic acids encoding aa 74 to 105 of the JDV Rev WT protein and associated with a putative NLS was amplified by PCR and cloned into the pEGFP-C1 vector as described above. Where indicated, alanine substitution mutants were introduced into the EGFP-JDV Rev WT protein by PCR site-directed mutagenesis using appropriate primers. To generate EGFP-GST and EGFP-GST-NLS, the GST-encoding sequence from pGEX-4T-1 (GE Healthcare, Mississauga, Ontario, Canada) was amplified with appropriate primers and cloned into pEGFP-C1 and pEGFP-NLS JDV, respectively, using the Gibson assembly mastermix (New England Biolabs, Ipswich, MA). The EGFP-βGal and EGFP-βGal-NLS constructs were similarly generated using the βGal-encoding sequence derived from pSV-βGal (Clontech). All constructs were validated by DNA sequencing through the McGill University Sequencing Services (Montréal, Québec, Canada).

### Fluorescence microscopy

Cells cultured on coverslips in 24-well cell culture plates were transfected with pEGFP-JDV Rev WT or each of the pEGFP-JDV Rev mutants. After an incubation of 24 h and, where indicated, 5 nM of leptomycin B (LMB), a known nuclear exit inhibitor [24], was added to the cell culture medium for 5 h. The cells were fixed with 4% paraformaldehyde in phosphate-buffered saline (PBS, pH 7.3) solution for 15 min. For the immunofluorescence assay, cells were permeabilized with 0.2% Triton X-100 for 10 min, blocked with 4% bovine serum albumin in PBS for 1 h at 37°C, and then incubated with rabbit polyclonal IgG primary anti-C23 (nucleolin) antibodies (H-250) (Santa Cruz Biotechnologies, Dallas, TX) for 1 h at 37°C. After three washes with PBS containing 0.2% Triton X-100, the cells were incubated with Alexa 647-labeled anti-rabbit secondary antibodies (Santa Cruz Biotechnologies) for 1 h at 37°C. Coverslips were mounted onto glass slides using ProLong Gold antifade reagent with 4,6-diamidino-2-phenylindole (DAPI) (Invitrogen, Carlsbad, CA). Cells were imaged by confocal laser scanning microscopy (CLSM) using a Nikon A1 confocal system, equipped with a 60× oil immersion objective. The NIH ImageJ 1.62 public domain software was used to determine the cytoplasmic (Fc), nuclear (Fn) and nucleolar fluorescence (Fno) values. These values were then utilized to determine the nuclear/cytoplasmic fluorescence ratio (Fn/c) by using the equation: Fn/c = (Fn − Fb)/(Fc − Fb), where Fb refers to the background fluorescence [25]. The same equation path was used to determine the nucleolar/nuclear fluorescence ratio (Fno/n). The data shown are representative of the expression patterns observed in 30 cells from three independent experiments (10 analyzed cells per experiment).

### CAT assay

The JDV Rev nuclear export activity was quantified in transient-transfection assays using a pDM138-based BIV Rev chloramphenicol acetyltransferase (CAT) reporter construct containing the BIV RRE (pRRE-BIV) [19]. HEK293T cells were seeded in 6-well cell culture plates and cotransfected with 0.5 μg of empty pEGFP-C1 or each of the pEGFP constructs encoding either the JDV Rev WT protein or each of the JDV Rev mutant proteins, and 0.5 μg of pRRE-BIV. Cells were harvested at 48 h after transfection and lysed with the lysis buffer [provided in the CAT enzyme-linked immunosorbent assay kit (CAT-ELISA kit; Roche, Penzberg, Germany)]. The amount of CAT in 50 μg of total cellular proteins was assessed using the CAT-ELISA kit. The CAT-ELISA data were normalized to the level of EGFP-JDV Rev protein expression as determined by Western blot [26]. The mean Rev activity value (obtained from three independent experiments, triplicate samples per experiment) of the JDV Rev WT and each of the JDV Rev mutant proteins was expressed as the mean ratio of respective EGFP-JDV Rev protein CAT expression to the basal expression of pRRE-BIV in presence of EGFP alone.

The pDM128 plasmid construct containing the HIV-1 RRE (pRRE-HIV) [27] was used in the CAT-ELISA to determine the activity of HIV-1 Rev and of all mutant proteins tested in the Rev(1.4)-EGFP nuclear export assay (see below). To this end, HEK293T cells were cotransfected with 0.5 μg of either empty pEGFP-C1, Rev(1.4)-NES3-EGFP or each of the Rev(1.4)-JDVNES-EGFP mutant constructs (see below) and 0.5 μg of pRRE-HIV. The HIV-1 Rev activity (obtained from three independent experiments, triplicate samples per experiment) was expressed as the mean ratio of Rev1.4-NES3-EGFP or mutant protein CAT expression to the basal expression of pRRE-HIV in presence of EGFP alone.

### SDS-PAGE and Western blot analysis

For each sample, a total cell extract quantity of 50 μg was separated by 12% sodium dodecyl sulfate-polyacrylamide gel electrophoresis (SDS-PAGE) and then electrotransferred onto nitrocellulose membranes. The membranes were blocked in PBS-Tween 0.05% (PBS-T) containing 5% of nonfat dry milk for 1 h at room temperature prior to the addition of mouse monoclonal primary antibodies specific to EGFP for 1 h (Santa Cruz Biotechnologies, B-2 clone). The membranes were then washed three times with PBS-T and incubated for 1 h at room temperature with anti-mouse horseradish peroxidase-conjugated IgGs (Thermo Fisher, Waltham, MA) diluted in PBS-T and 5% nonfat dry milk. The signal was detected by enhanced chemiluminescence with a Fusion FX7 apparatus (Vilber, Collégien, France). The bands were then analyzed with the ImageJ software to determine the EGFP-Rev expression level for CAT-ELISA data normalization.

### Rev(1.4)-EGFP nuclear export assay

To determine the aa important for Rev exportation, the Rev(1.4)-EGFP nuclear export assay was used [28]. This assay is based on the ability of a predicted NES sequence to promote the nuclear export of the HIV-1 NES-deficient Rev(1.4)-EGFP fusion protein. The NES-deficient Rev(1.4)-EGFP and Rev(1.4)-NES3-EGFP (a construct that contains the intact HIV-1 Rev NES sequence) plasmids were kindly provided by Dr Beric Henderson (University of Sydney, Sydney, Australia) [27]. Alanine substitution NES mutant sequences were derived from the predicted JDV Rev NES sequence by using complementary synthetic oligonucleotides that were ligated into the compatible ends of BamHI- and AgeI-digested Rev(1.4)-EGFP plasmid. All mutant constructs were validated by sequencing.

To conduct the Rev(1.4)-EGFP nuclear export assay, HEK293T cells were cultured on coverslips in 6-well cell culture plates. The cells (50% confluence) were then transfected with Rev(1.4)-EGFP (negative control), Rev(1.4)-NES3-EGFP (positive control), or plasmids containing the NES sequence of JDV Rev WT or each of the JDV Rev NES mutated sequences. After an incubation time of 24 h, transfected cells were left untreated or exposed to both cycloheximide (10 μg/ml) and actinomycin D (ActD) (5 μg/ml) for 3 h or to LMB (5 nM) for 1 h prior to the cycloheximide and ActD (cycloheximide/ActD) treatment. Cycloheximide is a protein synthesis inhibitor that was used to ensure that any cytoplasmic green fluorescence resulted from the nuclear export of EGFP fusion protein rather than novel synthesis of the protein. ActD was used to promote the re-localization of HIV-1 Rev in the cytoplasm [28]. At the end of the incubation period the cells were fixed, counterstained with DAPI, and the coverslips were mounted on glass slides using ProLong gold antifade reagent. Cells were imaged by CLSM and analyzed as described above. The data shown are representative of the expression pattern observed in 30 cells from three independent experiments (10 analyzed cells per experiment).

### Statistics

All the experimental results were expressed as mean values + the standard error about the mean (SEM). Statistical analysis was performed by using the GraphPad Prism 7 software (San Diego, CA). Unless otherwise specified, data were analyzed using either a Student’s T-test to compare data from two group means or, where applicable, corrected with the Holm-Sidak method for multiple comparison of the means, a one-way ANOVA followed by a post-hoc Dunnett’s test (ANOVA Dunnett’s test) to compare the mean of each of the JDV Rev mutants to that of the JDV Rev WT protein, or a one-way ANOVA followed by a post-hoc Tukey’s multiple-comparison test (ANOVA Tukey’s multiple-comparison test) to compare the means of the JDV Rev mutant proteins to each other’s.

## Results

### The JDV Rev protein fused to EGFP mainly localizes to the cytoplasm and nucleolus

**Fig 1:**
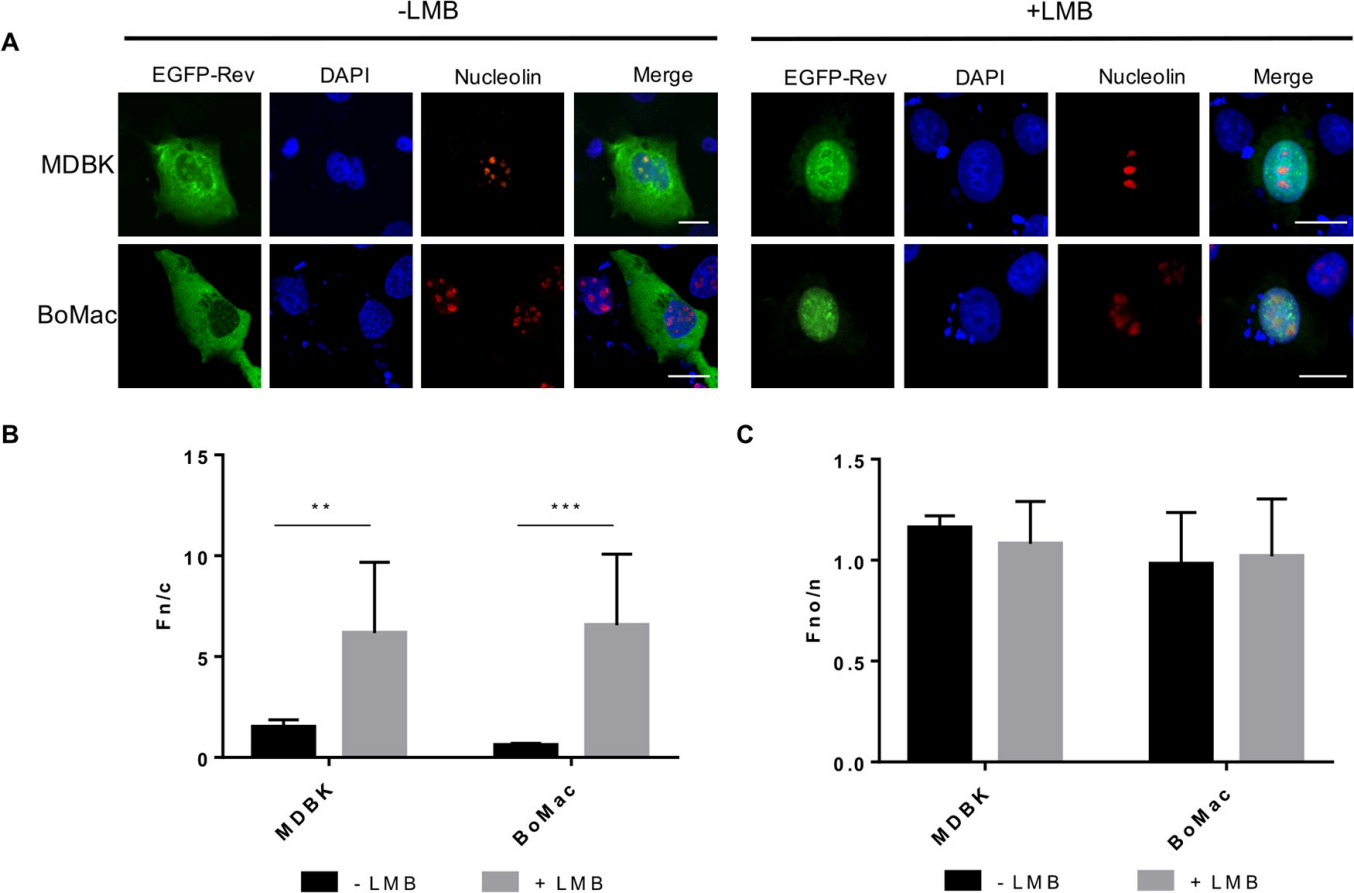
Subcellular localization of the JDV Rev WT protein fused to EGFP. **(A)** Microscopy analysis of EGFP-JDV Rev WT protein (in green) expressed in MDBK and BoMac cells following transfection for 24 h with pEGFP-JDV Rev WT. Cells were left untreated or treated with 5 nM of leptomycin B (LMB) for 5 h and then fixed, subjected to immunostaining for nucleolin detection (in red) and counterstained with DAPI for nucleus visualization (in blue). The merge panel represents the superposition of EGFP-JDV Rev, DAPI, and nucleolin images. The images shown are representative of the expression pattern observed in cells from three independent experiments. The white bars correspond to a length of 10 μm. Images were derived by using CLSM at 60x magnification and analyzed to determine the Fn/c **(B)** and the Fno/n **(C)** ratios. Results are expressed as the mean Fn/c or Fno/n ratio ± SEM (*n* = 30). For each cell line, significant differences, using a Student’s T-test corrected with the Holm-Sidak method for multiple comparison of the means, with and without LMB treatment, are indicated by ** (*P* < 0.005) and *** (*P* < 0.0005). No significant differences, using an ANOVA Tuckey’s multiple-comparison test, were observed between the Fn/c and Fno/n ratios measured for the MDBK and BoMac cells, regardless of the LMB treatment.

To determine the intracellular localization of the JDV Rev protein, bovine cell lines (MDBK and BoMac) were transfected with pEGFP-JDV Rev WT, encoding the JDV Rev WT protein fused to EGFP. In MDBK and BoMac transfected cells, the EGFP-JDV WT Rev protein predominantly localized to the cytoplasm of bovine cells without LMB treatment (Fig 1A and 1B) with a slight nucleolar localization (Fig 1A and 1C), as expected for a lentiviral Rev protein [17, 19]. Moreover, the Rev protein colocalized with nucleolin used as a nucleolar marker (Fig 1A). No significant differences were observed between the Fn/c and Fno/n ratios measured for the MDBK and BoMac cells. Considering that transfection of MDBK cells resulted in a higher number of cells expressing EGFP-JDV Rev WT, subsequent experiments were conducted using these cells.

### The export of the JDV Rev protein from the nucleus to the cytoplasm involves the CRM1 protein pathway

All Rev and Rev-like proteins characterized so far interact with the CRM1 protein, allowing them to be exported from the nucleus to the cytoplasm [29, 30–33]. To determine whether the JDV Rev WT export is dependent on the CRM1 protein pathway, the BoMac and MDBK cells were transfected each with pEGFP-JDV Rev WT for 24 h and then treated for 5 h with LMB, a well-known inhibitor of CRM1. As shown in Fig 1A and 1B, the JDV Rev WT protein accumulated exclusively in the nucleus of bovine cells in presence of LMB, indicating that the JDV Rev WT nucleocytoplasmic export is CRM1-dependent. These results obtained with these cells also indicated that the cellular distribution of JDV Rev WT was not a cell-line specific effect.

It is noteworthy that the Fno/n ratios obtained for all LMB-treated bovine cells were not significantly different from those of LMB-untreated cells even though the Rev protein accumulated in the nucleus. These results were attributed to the assumption that nucleus accumulation of Rev more likely lead to more proteins in the nucleolus such as the Fno/n ratios were kept similar in presence or absence of LMB (Fig 1C).

### Subcellular localization of the JDV Rev deletion mutant proteins

**Fig 2:**
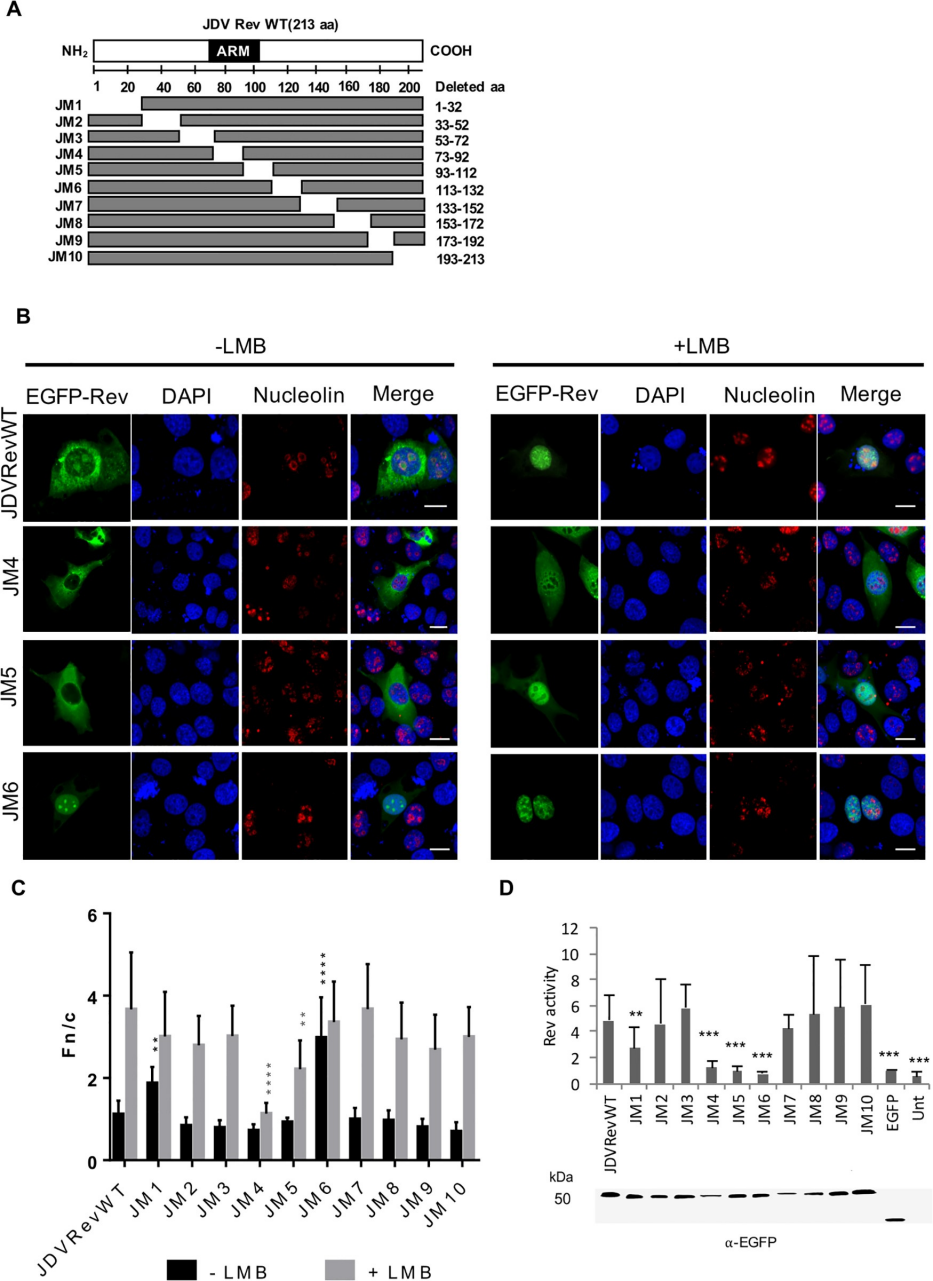
Subcellular localization of the JDV Rev deletion mutant proteins fused to EGFP. **(A)** JDV Rev mutant-encoding sequences were generated from the JDV Rev WT gene by PCR-ligation-PCR and then cloned into pEGFP-C1 for expression of the JDV Rev mutant (JM) proteins fused to EGFP. ARM: arginine-rich motif. **(B)** Microscopic analysis of the JM4 to JM6 mutant proteins (in green) expressed in MDBK cells at 24 h post transfection. Cells were fixed, subjected to immunostaining for nucleolin detection (in red) and counterstained with DAPI for nucleus visualization (in blue). Images were derived by using CLSM at 60x magnification and are representative of the expression pattern observed in cells from three independent experiments. The merge panel represents the superposition of EGFP-JDV Rev, DAPI, and nucleolin images. The while bars correspond to a length of 10 μm. CLSM images were analyzed to determine the Fn/c ratios (**C**) without or with LMB treatment. Results expressed as the mean Fn/c ratio ± SEM (*n* = 30) are shown for the JDV Rev WT protein and each of the JDV Rev deletion mutants. Significant differences, using an ANOVA Dunnett’s test, between the JDV Rev WT protein and each of the deletion mutant proteins (JM1 to JM10) are indicated by ** (*P* < 0.005). **** (*P* < 0.00005). **(D)** The nuclear export activity of EGFP-JDV Rev WT or EGFP-Rev deletion mutant proteins (JM1 to JM10) was determined using a CAT reporter assay. The CAT expression levels were normalized to EGFP-Rev expression for each protein in cell lysates as determined by Western blot using an EGFP-specific antibody (bottom of the panel). Rev activity was then determined as the ratio of EGFP-JDV Rev WT or mutant protein CAT expression to the basal expression from pDM128 or pDM138 constructs co-transfected with empty pEGFP-C1 only. The mean Rev activity values + SEM were obtained from three independent experiments (triplicate samples per experiment). Significant differences, using an ANOVA Dunnett’s test, between the JDV Rev WT protein and each of the deletion mutant proteins are indicated by ** (*P* < 0.005), and *** (*P* < 0.0005). Unt: untransfected cells.

In order to identify the region necessary for nuclear and nucleolar localization, plasmids encoding for a series of JDV Rev deletion mutants fused to EGFP were generated (Fig 2A), and the subcellular distribution of the proteins was analyzed by fluorescence microscopy in presence or absence of LMB. The results showed that seven of the ten Rev mutants (JM1, JM2, JM3, JM7, JM8, JM9 and JM10) showed a subcellular distribution similar to that of the JDV Rev WT protein with a cytoplasmic and nucleolar localization without LMB treatment, and a predominant nuclear localization in presence of LMB (S1 Fig). The JM1 mutant localized to the cytoplasm as the mutants described above in absence of LMB. However, it also showed a prominent nuclear localization as compared to the JDV REV WT protein in absence of LMB treatment (Fig 2C and S1 Fig). This observation was attributed to the deletion in that mutant of the R-D-L-L-Q-R-A-V sequence that is similar to the E-D-L-L-R-A-V motif present in the HIV-1 Rev first multimerization domain [34]. Indeed, preliminary data obtained in our laboratory identified the deleted sequence in JM1 to be associated with the multimerization domain of the JDV Rev WT protein. Considering that a lentivirus Rev protein must at least dimerize to interact with CRM1, thereby allowing its export from the nucleus to the cytoplasm, the deleted sequence in JM1 would have led to a reduced export and, thereof, nuclear accumulation of the mutant protein [34]. In contrast, the JM4 mutant showed a strong cytoplasmic signal with a minor nuclear localization in presence of LMB (Fig 2B and 2C). This result indicated that the deleted sequence in mutant JM4 contains aa associated with NLS function. In addition, mutant JM4 showed a weak nucleolar signal, thereby indicating that the sequence deleted in this mutant also has an impact on the nucleolar localization of JDV Rev (Fig 2B and 2C). The JM5 mutant localized to the cytoplasm and nucleus of transfected cells but poorly in the nucleoli in absence of LMB (Fig 2B). This result suggested that the sequence deleted in mutant JM5 contains aa associated with NoLS function. Finally, the JM6 mutant only accumulated in the nucleus of transfected cells regardless of the LMB treatment (Fig 2B and 2D). This indicated that the sequence deleted in mutant JM6 more likely contains a NES. Also, for all proteins but JM6, the difference between the Fn/c ratios without LMB and with LMB was significant according to a Student’s T-test corrected with the Holm-Sidak method for multiple comparison of the means.

After determining the subcellular localization of JDV Rev WT and each of the Rev mutant proteins, the impact of the mutations on their nuclear export activity was examined by conducting a RNA export assay using the pRRE-BIV CAT reporter construct. This vector contains the CAT gene and the BIV RRE derived from the *env* gene [19, 35] within an intron flanked by HIV-1 splice sites [36]. Unspliced transcripts from the pRRE-BIV CAT construct are exported to the cytoplasm only if functional Rev is present, resulting in a significant CAT activity. In absence of functional Rev, only background CAT activity can be detected. As the JDV RRE sequence was still not characterized at the time of this study, a preliminary experiment was conducted to assess the nuclear export activity of JDV Rev using heterologous BIV and HIV-1 RRE [27]. Since a significant result was only obtained by using the BIV RRE sequence (S2 Fig), all further JDV Rev activity assays were conducted using pRRE-BIV CAT construct. As shown in Fig 2D, mutants JM1, JM4, JM5, and JM6 displayed an export activity that was significantly lower than that of the JDV Rev WT protein. The results obtained with mutants JM4 and JM6 are consistent with, as mentioned above, the absence of NLS or NES in these mutants, respectively. The low CAT activity level obtained for mutant JM5 may be attributed to residues deleted in this sequence that might be part of the NLS or, alternatively, of the RNA binding domain (RBD) necessary to interact with the RRE. Altogether, these results suggest that the sequence deleted in mutants JM4 and JM5 contains residues important for the nuclear and/or nucleolar localization of the JDV Rev protein. Finally, the decrease in Rev activity for mutant JM1 was attributed to the loss of a multimerization domain-associated NIS-like sequence the counterpart of which, in HIV-1, has been reported to have a negative impact on the virus replication [34]. In any case, the region deleted in mutant JM1 does not contain a NLS, as the protein readily localized to the nucleus.

### The sequence encompassing amino acids 74 to 105 of the JDV Rev protein directs the nuclear/nucleolar localization of chimeric proteins

**Fig 3:**
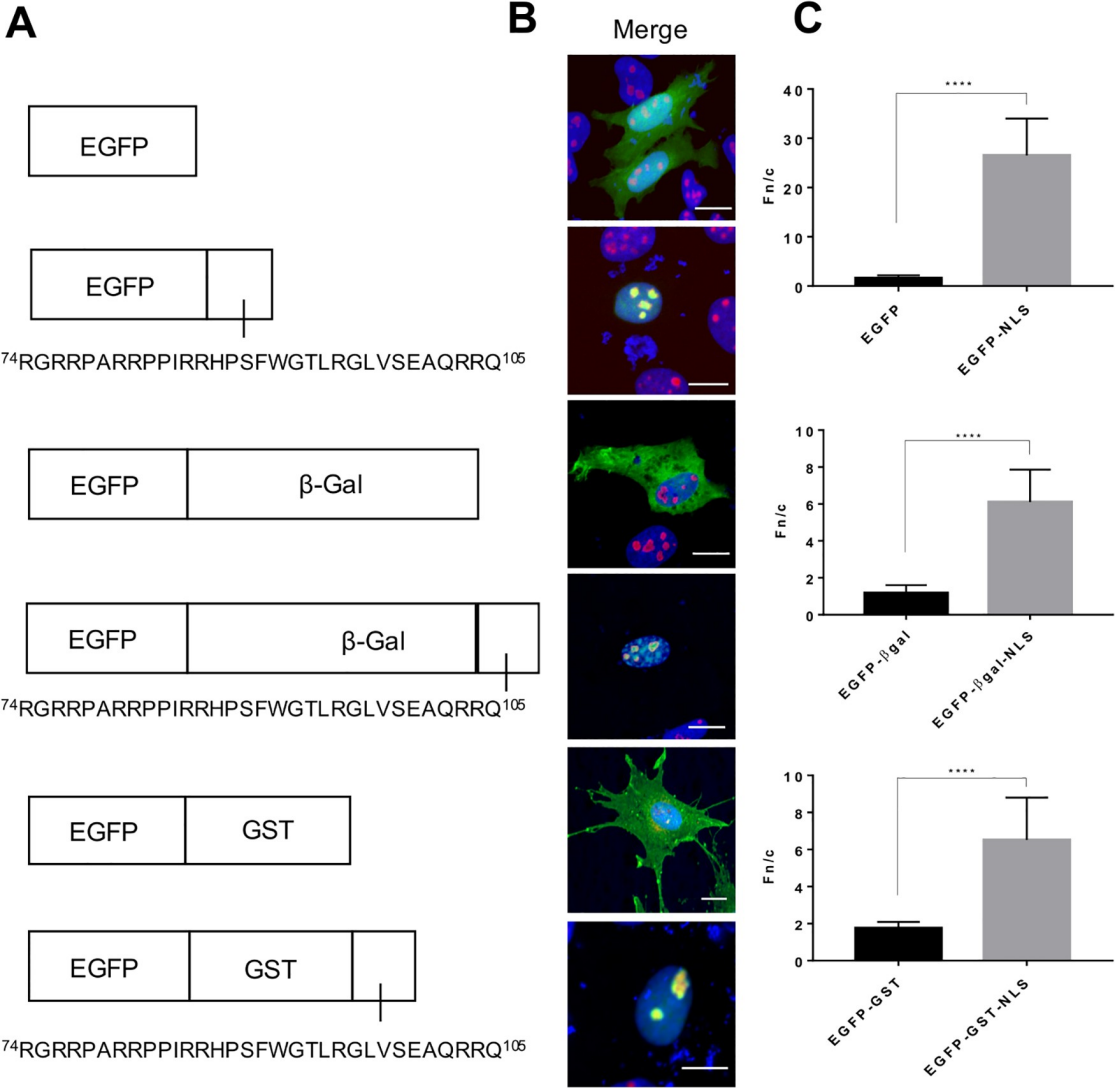
The region encompassing amino acids (aa) 74 to 105 of the JDV Rev protein is associated with NLS/NoLS functions. **(A)** The 74 to 105 aa sequence containing basic residues present in deleted sequences of JDV Rev mutants JM4 and JM5 was inserted into pEGFP-C1, pEGFP-βGal or pEGFP-GST vectors. **(B)** MDBK cells were transfected with plasmid vectors encoding either the EGFP, EGFP-βGal or EGFP-GST proteins alone or fused to the JDV 74-105_REV_ sequence described in panel A. After an incubation time of 24 h, the cells were fixed, subjected to immunostaining for nucleolin detection (in red) and counterstained with DAPI for nucleus visualization (in blue). Expression of the proteins was detected via the EGFP fluorescence (in green). Images were derived by using CLSM at 60x magnification and are representative of the expression pattern observed in cells from three independent experiments. The merge panel represents the superposition of EGFP, DAPI and nucleolin images. The white bars correspond to a length of 10 μm. **(C)** CLSM images were analyzed to determine the Fn/c ratios. Results (mean Fn/c ratio ± SEM, for *n* = 30) are shown for the different EGFP proteins. Significant differences, using a Student’s T-test, between EGFP, EGFP-GST or EGFP-βGal and each of the protein counterparts fused to the JDV 74-105_REV_ sequence, are indicated by ****(*P* < 0.00005).

The trafficking of molecules from the cytoplasm to the nucleus and nucleolus is mediated by NLS and NoLS, respectively. These signals are usually composed of basic aa like arginines and lysines [37–39]. Consequently, the basic aa contained in the deleted sequences of mutants JM4 and JM5 could be associated with NLS and/or NoLS functions. To examine this, the sequence composed of aa 74 to 105 and rich in arginine residues was fused to the C terminus of EGFP (Fig 3A). Expectedly, EGFP (~27 kDa) alone showed diffuse distribution in the cytoplasm and nucleus/nucleolus of transfected cells (Fig 3B). In contrast, the EGFP 74-105_REV_ chimeric protein accumulated in the nucleus and in the nucleolus and was totally absent from the cytoplasm. To confirm that the nuclear localization was attributed to the presence of a functional NLS and not to the diffusion followed by retention of the protein in the nucleus due to a nuclear retention signal, a cytoplasmic protein of larger size, namely EGFP-βGal (~143 kDa as a monomer and ~560 kDa in the tetrameric active form of βGal) fused to the JDV NLS sequence, was used. EGFP-βGal localized almost exclusively to the cytoplasm. In contrast, EGFP-βGal-NLS JDV protein localized mainly in the nucleus/nucleolus of transfected cells (Fig 3A and 3B). Essentially identical results were obtained when the JDV NLS was fused to a protein of lesser size (EGFP-GST, ~60 kDa). Altogether the results unequivocally indicated that the sequence encompassing aa 74 to 105 of the JDV Rev WT protein contains functional NLS/NoLS that can direct cytoplasmic proteins, in this case EGFP, EGFP-βGal and EGFP-GST, to the cell nucleus and nucleolus.

### The NLS motif in the JDV Rev protein is monopartite-like

**Fig 4:**
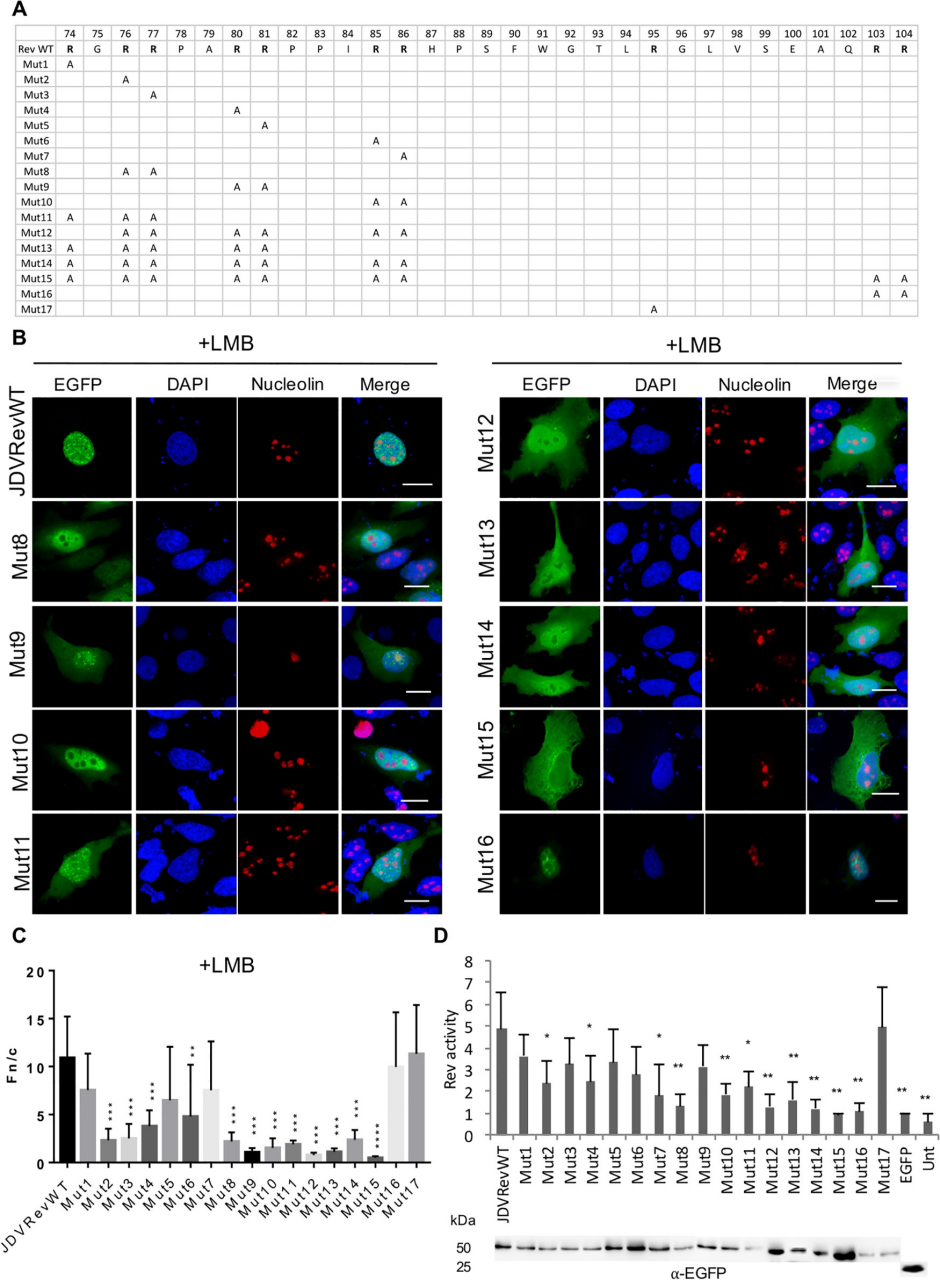
The NLS of the JDV Rev protein is monopartite-like. **(A)** JDV Rev arginine (R) to alanine **(A)** substitution mutant proteins (Mut1 to M17) were generated from pEGFP-JDV Rev WT. **(B**) Subcellular localization of JDV Rev WT and mutant proteins harboring multiple arginine to alanine substitutions (Mut8 to Mut16) described in panel A. MDBK cells were transfected with pEGFP-JDV Rev WT or each of the mutant constructs, and incubated for 24 h. Cells were treated with leptomycin B (LMB) for 5 h or left untreated. Cells were then fixed, subjected to immunostaining for nucleolin detection (in red) and counterstained with DAPI for nucleus visualization (in blue). Images were derived by using CLSM at 60x magnification and are representative of the expression pattern observed in cells from three independent experiments. The merge panel represents the superposition of EGFP, DAPI and nucleolin images. The white bars correspond to a length of 10 μm. **(C)** CLSM images were analyzed to determine the Fn/c ratios. Results (mean Fn/c ratio ± SEM, for *n* = 30) are shown for the JDV Rev WT protein and each of the alanine substitution mutants. Significant differences, using an ANOVA Dunnett’s test, between the JDV Rev WT protein and each of the substitution mutant proteins with LMB treatment are indicated by ** (*P* < 0.005), *** (*P* < 0.0005), and **** (*P* < 0.00005). **(D)** Nuclear export activity of the EGFP-JDV Rev WT and mutant proteins (Mut1 to Mut17) was determined using a CAT reporter assay. The CAT levels were normalized to the expression level of EGFP-JDV Rev WT or mutant proteins as determined by Western blot using an EGFP-specific antibody (bottom of the panel). Rev activity was then determined as the ratio of EGFP-JDV Rev WT or mutant protein CAT expression to the basal expression from pDM128 or pDM138 constructs co-transfected with empty pEGFP-C1 only. The mean Rev activity values + SEM were obtained from three independent experiments (triplicate samples per experiment). Significant differences, using an ANOVA Dunnett’s test, between the JDV Rev WT protein and each of the substitution mutant proteins are indicated by * (*P* < 0.05) and ** (*P* < 0.005). Unt: untransfected cells.

To identify which aa are necessary for the NLS function of JDV Rev, a series of mutants (Mut1 to Mut17) were generated by site-directed mutagenesis in which arginine (R) residues located in the 74–105 aa sequence described above, were successively substituted with alanine (A) residues (Fig 4A). Single alanine substitutions at residues 74 (Mut1), 81 (Mut5), 86 (Mut7), or 95 (Mut17) had no impact on the cell distribution of the mutant proteins as they showed, as for the JDV Rev WT protein, a predominant nuclear localization in presence of LMB (Fig 4B and S3 Fig). In contrast, alanine substitutions at residues 76 (Mut2), 77 (Mut3), 80 (Mut4), and 85 (Mut6) had a negative effect on the localization of these mutant proteins in presence of LMB as they were cytoplasmic and nuclear (Fig 4C and S3 Fig). Double alanine substitutions at residues 76–77 (Mut8), 80–81 (Mut9) and 85–86 (Mut10), and combined alanine substitutions (Mut11 to Mut15) targeting a series of arginine residues from aa 74 to 86 also had a somewhat more negative effect on the nuclear localization of these mutant proteins in presence of LMB as determined by their low Fn/c ratios (Fig 4B and 4C). Finally, mutant Mut16 with double alanine substitutions at residues 103 and 104 showed a cell distribution phenotype similar to that of the JDV Rev WT protein with a strong nuclear localization in presence of LMB, indicating that these residues are not associated with NLS function. It is noteworthy, however, that this mutant protein did not localize to the cell nucleolus regardless of the LMB treatment (S3 Fig), suggesting that the 103 and 104 arginine residues are associated with NoLS function. Combined, the results obtained from the JDV Rev alanine substitution mutants are indicative of the presence of a monopartite-like NLS in JDV Rev that is composed of residues ^76^RRPARRPPIRR^86^. However, as a so-called monopartite NLS is defined as a classical NLS [15, 16], importin α-binding experiments [14] are needed to unequivocally confirm the monopartite nature of JDV Rev NLS. Therefore, the monopartite-like NLS designation was used herein.

As for the JDV Rev deletion mutants, the impact of the mutations on the nuclear export activity of mutants Mut1 to Mut17 was assessed using the RNA export CAT assay described above. As shown in Fig 4D, mutants Mut2, 4, 7, 8, and 10 to 16 showed a significant decrease in their export activity when compared to that of the JDV Rev WT protein. These results correlated with the distinct cell distribution of all these mutants but Mut2, Mut4 and Mut7 when compared to that of the JDV Rev WT protein (Fig 4C). Mutant Mut16 that was able to enter the nucleus but not the nucleolus, regardless of the LMB treatment, also displayed a significant decrease in nuclear export activity when compared to that of JDV Rev WT (*P* < 0.005). Finally, mutant Mut17 had a nuclear export activity similar to that of the JDV Rev WT protein.

#### The NoLS of the JDV Rev protein is bipartite in structure

**Fig 5:**
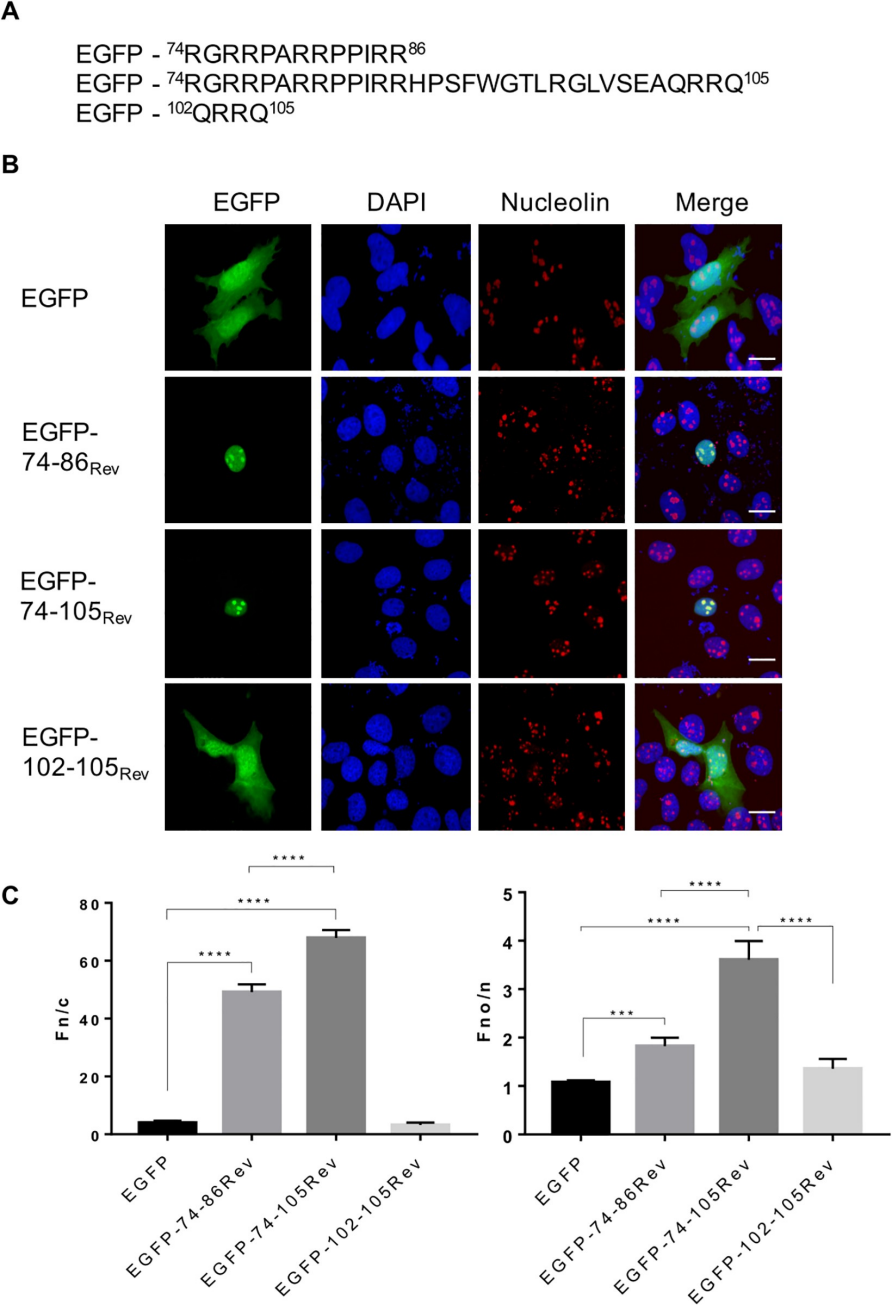
Subcellular localization of EGFP fused to JDV Rev arginine residue cluster sequences. **(A)** MDBK cells were transfected with plasmid constructs encoding either EGFP or EGFP fused, at its C-terminal, to each of the JDV Rev arginine cluster sequences shown in the figure. **(B)** After an incubation of 24 h the cells were fixed, subjected to immunostaining for nucleolin detection (in red) and counterstained with DAPI for nucleus visualization (in blue). Images were derived by using CLSM at 60x magnification and are representative of the expression pattern observed in cells from three independent experiments. The merge panel represents the superposition of EGFP, DAPI and nucleolin images. The white bars correspond to a length of 10 μm. **(C)** The CLSM images were analyzed to determine the Fn/c and Fno/n ratios. Results were expressed as the mean Fn/c or Fno/n ratio ± SEM (*n* = 30). Significant differences between the EGFP proteins, using an ANOVA Tukey’s multiple-comparison test, are indicated by *** (*P* < 0.0005) and **** (*P* < 0.00005).

To identify the aa important for the nucleolar localization of the JDV Rev WT protein, the mutants described above (Mut1 to Mut17) were used to transfect the MDBK cells in absence or presence of LMB. As shown in S4 Fig, the Fno/n ratios of all mutant proteins but Mut17 were significantly lower than that of the JDV Rev WT protein, suggesting that the arginine residues substituted in these mutant proteins are important for the nucleolar localization of JDV Rev. Assuming that mutations affecting in the first place the nuclear import of a given protein will also influence its nucleolar localization, we wished to address the above JDV Rev NoLS composition assumption by an independent means. Thus the 74–86, 74–105 and 102–105 aa sequences of the JDV Rev WT protein, described in Fig 5A, were fused successively to EGFP, a protein that can reach the nucleus by passive diffusion. As found in Fig 5B and 5C, the 74–86 cluster of arginine residues was sufficient to localize EGFP to both the nucleus and nucleolus. In contrast, the 102–105 arginine residue cluster had no effect on EGFP localization. However, as shown in Fig 5C, the complete 74–105 sequence showed stronger nuclear and nucleolar accumulation of EGFP than the 74–86 sequence indicating that both arginine clusters (74–86 and 102–105) indeed are important for optimal NoLS function. Based on these results and on the fact that mutant Mut16 of the JDV Rev protein, as mentioned above, was excluded from the nucleolus (Fig 4), we concluded that the NoLS of JDV Rev is bipartite in structure, being composed of arginine residues present in the ^74^RGRRPARRPPIRR^86^ and ^102^QRRQ^105^ aa sequences.

#### The NES of the JDV Rev protein

The canonical NES is generally composed of a short leucine-rich region, although other hydrophobic aa like methionine, phenylalanine and valine have also been identified in NES sequences [40, 41]. As shown in Fig 2B, the JDV Rev JM6 mutant protein accumulated in the nucleus of transfected cells regardless of the LMB treatment. Moreover, it was unable to export RNA from the nucleus to the cytoplasm in contrast to the JDV Rev WT protein as shown in the Rev activity assay (Fig 2E). Accordingly, it was hypothesized that the region spanning from aa 113 to 132 likely contains a NES. This postulate was supported by using the NetNES 1.1 prediction program [42], which identified a putative NES CRM1-dependent signal from aa 116 to 129 in the JDV Rev WT protein sequence.

**Fig 6:**
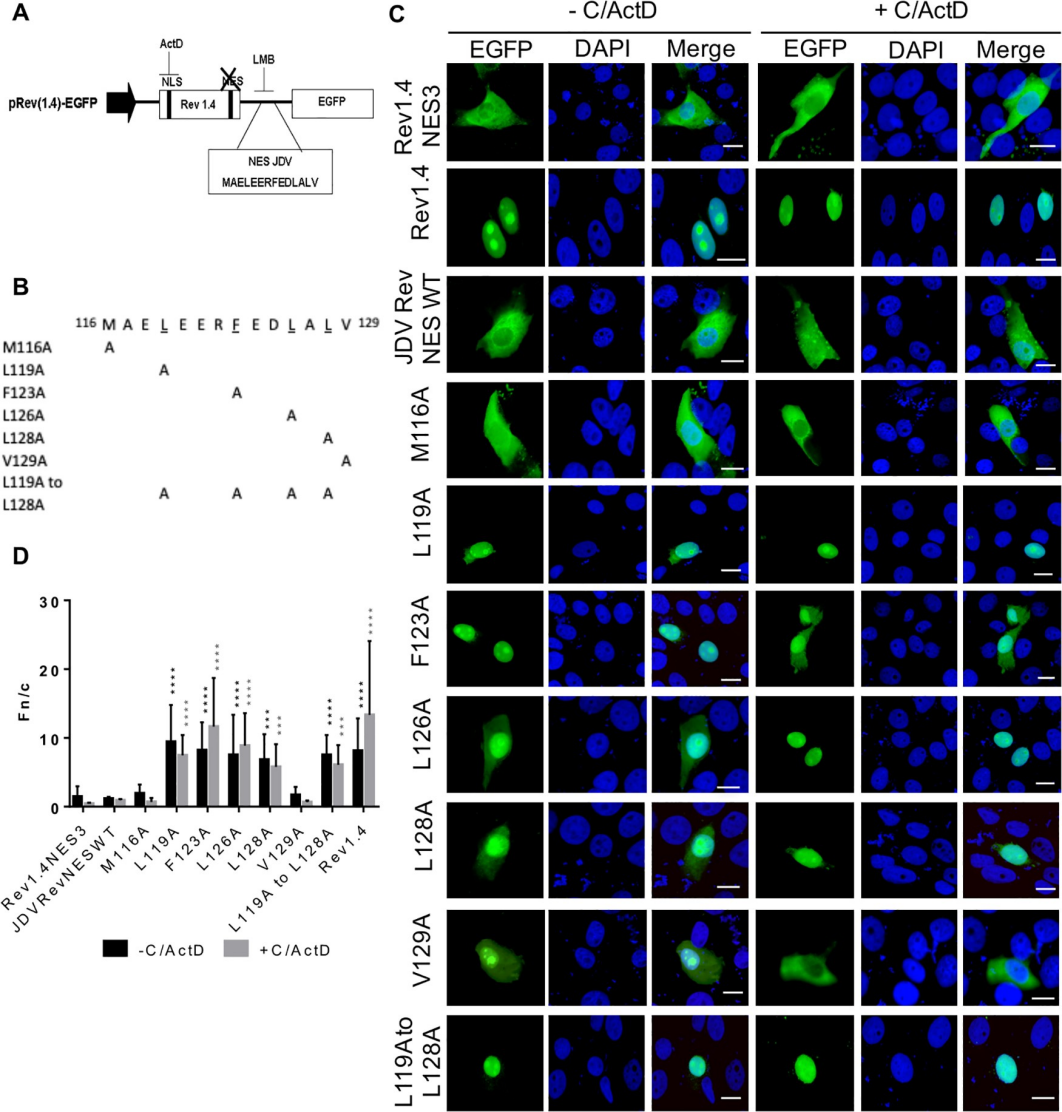
The JDV Rev protein contains a nuclear export signal (NES). **(A)** Plasmids encoding HIV-1 Rev(1.4)-EGFP (negative control; Rev1.4), HIV-1 Rev(1.4)-NES3-EGFP (positive control; Rev1.4NES3), or plasmids encoding HIV-1 Rev(1.4) containing either the predicted NES sequence (amino acids 116 to 129) of the JDV Rev WT protein (JDVRevNESWT) or **(B)** each of the JDV NES mutated sequences were used. (**C**) HEK293T cells were transfected and, after an incubation of 24 h, were either left untreated (-) or exposed (+) to both cycloheximide and actinomycin D (C/ActD). Cells were fixed and counterstained with DAPI for nucleus visualization (in blue). **(D)** Images were derived by using CLSM at 60x magnification and are representative of the expression pattern observed in cells from three independent experiments. The merge panel represents the superposition of EGFP and DAPI images. The white bars correspond to a length of 10 μM. CLSM images were analyzed to determine the Fn/c ratios. Results (mean Fn/c ratio ± SEM, for *n* = 30) are shown for the different proteins. Significant differences, using an ANOVA Dunnett’s test, in presence or absence of C/ActD, between the JDVRevNESWT-containing HIV-1 Rev(1.4) protein and the HIV-1 Rev(1.4) protein containing each of the JDV Rev NES mutated sequences are indicated by *** (*P* < 0.0005) and **** (*P* < 0.00005).

In order to identify residues important for the export of JDV Rev from the nucleus to the cytoplasm, the HIV-1 Rev(1.4)-EGFP nuclear export assay was used [27]. In this assay, the NES is evaluated by analyzing its capacity to promote nuclear export of the HIV-1 Rev(1.4)-EGFP fusion protein deficient in NES function. Inserting a functional NES sequence in the pRev(1.4)-EGFP vector between the HIV-1 Rev(1.4) and EGFP sequences (as indicated in Fig 6A) restores the nucleocytoplasmic shuttling activity of the fusion protein.

**Fig 7:**
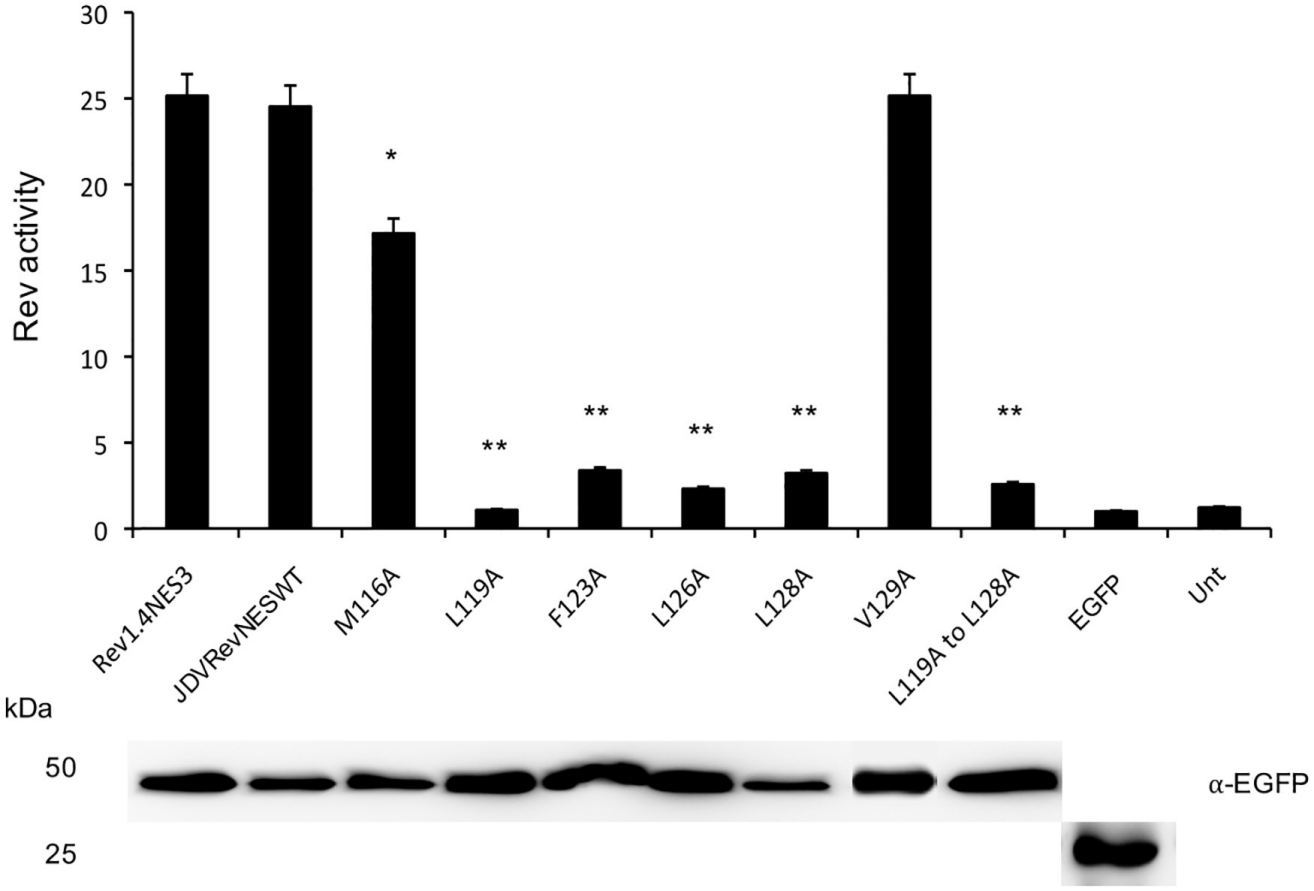
The nuclear export activity of HIV-1 Rev containing JDV Rev WT or mutated NES sequences. The nuclear export activity of the EGFP-fused HIV-1 Rev proteins described in Fig 6 was determined using a CAT reporter assay. The CAT expression data were normalized to the expression level of the proteins as determined by Western blot using an EGFP-specific antibody (bottom of the figure). Rev activity was then determined as the ratio of HIV-1 Rev protein CAT expression harboring the HIV-1 Rev NES WT (NES3), the JDV Rev NES WT or the JDV Rev NES mutant to the basal expression from pDM128 construct co-transfected with empty pEGFP-C1 only. The mean Rev activity values + SEM were obtained from three independent experiments (triplicate samples per experiment). Significant differences, using an ANOVA Dunnett’s test, between the JDV Rev WT NES and each of the JDV Rev NES mutant proteins are indicated by * (*P* < 0.05) and ** (*P* < 0.005). Unt: untransfected cells.

The predicted NES sequence (aa 116 to 129) of JDV Rev was inserted into the pRev(1.4)-EGFP vector (Fig 6A) as well as sequences containing various single alanine substitutions targeting hydrophobic aa (methionine, leucine, phenylalanine and valine) of the putative JDV Rev NES (Fig 6B). HEK293T cells were transfected with each of the construct and, after an incubation of 24 h, were left untreated or treated with cycloheximide/ActD for 3 h. To evaluate the strength of each of the inserted JDV Rev NES sequences (WT or mutated), the Fn/c ratios were calculated. As expected, the HIV-1 Rev(1.4)-EGFP protein (Rev1.4) lacking a functional NES exclusively localized to the nucleus and nucleolus of transfected cells in presence of cycloheximide/ActD with a Fn/c ratio of 13.35 (Fig 6C). In contrast, the HIV-1 Rev(1.4)-NES3-EGFP protein (Rev1.4-NES3) which contains an intact HIV-1 Rev NES, localized mainly to the cytoplasm of cycloheximide/ActD-treated cells with a Fn/c ratio of 0.58. Insertion of the JDV Rev WT predicted NES sequence within the vector resulted in the localization of the HIV-1 Rev-EGFP protein (JDVRevNESWT) in the cytoplasm of the transfected cells treated with cycloheximide/ActD with a resulting Fn/c ratio of 0.91. Similarly, low Fn/c ratio results were obtained with the M116A and V129A mutants, suggesting that both M116 and V129 residues have no role in NES function of JDV Rev. In contrast, all the other single substitutions in the JDV NES sequence had an impact on the subcellular localization of the HIV-1 Rev. The L119, F123A, L126A and L128A mutations had a negative impact on the export of the protein with Fn/c ratios of 7.45, 11.64, 8.86 and 5.78, respectively (Fig 6C and 6D). All these results were confirmed by the RNA export assay using the pRRE-HIV CAT reporter construct (pDM128) [27]. Indeed the HIV-1 Rev export activity was significantly lower for mutants L119A, F123A, L126A and L128A when compared to that of the HIV-1 Rev proteins containing either the predicted sequence of JDV Rev NES (JDVRevNESWT) or the intact HIV-1 Rev NES sequence (Rev1.4NES3) used as a positive control (*P* < 0.005) (Fig 7). Moreover, the M116A mutation affected negatively the export activity of HIV-1 Rev (*P* < 0.05). Altogether, these results showed that residues ^119^L, ^123^F, ^126^L, and ^128^L are mandatory for the JDV Rev NES function, whereas the residue ^116^M also impacts this function.

## Discussion

The Rev and Rev-like proteins of complex retroviruses/lentiviruses are regulatory proteins that mediate the nucleocytoplasmic transport of viral RNAs. To exert their function, the Rev proteins shuttle between the nucleus and cytoplasm through the involvement of import and export cell receptors that interact with the Rev NLS and NES, respectively. Moreover, lentiviruses like HIV-1 and BIV, and presumably all other lentiviruses, target the Rev protein to the nucleolus via NoLS [17, 19].

This study reports for the first time the subcellular distribution of the JDV Rev protein and the localization of the NLS, NoLS and NES in the protein. As mentioned above, no *in vitro* cell systems permissive for JDV replication have been reported so far. Consequently, the subcellular distribution experiments were conducted in cells transiently transfected with appropriate JDV Rev-encoding plasmid constructs, as previously described by us to characterize in detail the BIV Rev protein [14, 19]. Here it was first demonstrated that the JDV Rev protein fused to EGFP localized predominantly to the cytoplasm and the nucleolus (Fig 1). This result is similar to that described for HIV-1 Rev [43], but contrasts with the BIV Rev subcellular localization, which is predominantly nuclear/nucleolar [19]. By using several deletion and alanine substitution mutants we identified the aa region necessary for the localization of JDV Rev in the nucleus. The subcellular localization of both JM4 and JM5 deletion mutants contrasted with the cytoplasmic and nucleolus distribution of JDV Rev WT. These two proteins were distributed mostly in the cytoplasm for mutant JM4 with a faint presence in the nucleus, or in the cytoplasm/nucleus but not in the nucleoli for mutant JM5. In addition, the Rev nuclear export activity of both mutants JM4 and JM5 was completely abolished in the CAT reporter assay (Fig 2). These results indicated that the sequences deleted in mutants JM4 and JM5 (aa 73 to 92 and aa 93 to 112, respectively) were important for the subcellular localization and biological activity of JDV Rev. The sequences deleted in mutants JM4 and JM5 were then analyzed and revealed the presence of a region rich in basic residues, essentially arginines, from aa 74 to 105 that would be potentially associated with NLS/NoLS functions. This assumption was confirmed when this region fused to the C-terminus of EGFP, EGFP-βGal or EGFP-GST relocated these proteins to the nucleus and nucleolus (Fig 3). In order to identify which basic aa were at play for the NLS function of JDV Rev, a series of point mutations were generated within the 74 to 105 aa region of the protein targeting the arginine (R) residues. The results obtained with Mut11 to 14 mutants showed that the ^76^RR-RR-RR^86^ aa cluster undoubtedly was important and sufficient for the NLS function of JDV Rev. This indicated that the NLS of JDV Rev has a monopartite-like structure and is composed of ^76^RR-RR-RR^86^. Thus, the JDV Rev NLS differs from the bipartite structure of BIV Rev NLS but resembles that of HIV-1, as it is composed exclusively of arginine residues (Table 1).

**Table 1:** Retroviral Rev and Rev-like NLS/NoLS sequences.

Protein^1^	NLS/NoLS^2^	Reference
HIV-1 Rev	RQARRN**RRRR**WRERQRQ	[17]
EIAV Rev	**KRRRK**	[44]
MMTV Rem	AL**RRKRRR**EMRK	[33]
HERV-K Rec	**RRRR**HRNRAP	[45]
HTLV-1 Rex	P**K**T**RRR**P**RR**SQ**RKR**PPTP	[17]
BIV Rev	**R**A**RK**LPGERRPGFWKSLRELVEQN**R****RK**QE**RR**	[19]
JDV Rev	RG**RR**PA**RR**PPI**RR**HPSFWGTLRGLVSEAQRRQ	This study

^1^Abbreviations: HIV-1, human immunodeficiency virus type 1; EIAV, equine infectious anemia virus; MMTV, mouse mammary tumor virus; HERV-K, human endogenous retrovirus K; HTLV-1, human T-lymphotropic virus 1; BIV, bovine immunodeficiency virus; JDV, Jembrana disease virus.

^2^The basic residues associated with NLS are shown in bold; the NoLS residues are underlined.

The nucleolus is the largest subnuclear structure that comprises the fibrillar center, the dense fibrillar center (DFC) and the granular component (GC) [46]. Beside its well-known role in rRNA synthesis, the nucleolus can play a role in replication of certain viruses [47]. Indeed, viruses may encode proteins that enter the nucleus and/or nucleolus and then interact with different partners to allow or improve steps of the viral replication cycle [48]. For instance, the Rev protein of BIV and HIV-1 were demonstrated to interact with the B23 nucleolar protein and this interaction was shown to be critical in the replication of these viruses [49, 50].

The targeting of a protein to the nucleolus relies on the presence of a NoLS that can be monopartite, bipartite or tripartite in structure [38, 51–53] or, in the absence of the latter, on interactions with nucleic acids or other NoLS-containing proteins [46]. Herein it was shown that the JDV Rev protein colocalized with the nucleolar marker nucleolin, known to be present in both GC and DFC nucleolus regions [38]. This nucleolar localization was dependent on the presence of a NoLS that was shown to be composed of the arginine residue at position 74 (^74^R), in addition to the ^76^RR-RR-RR^86^ and ^103^RR^104^ arginine residues. Therefore, the JDV Rev NoLS is bipartite in structure and differs from the NLS, intrinsically associated-monopartite structure of the NoLSs of HIV-1 Rev and Human T cell leukemia virus Rex proteins, and from the BIV Rev NoLS present in the spacer sequence that delimits the two BIV Rev NLS motifs [17]. Combined together, the JDV Rev protein contains a novel type of NoLS among previously described retroviral Rev or Rev-like proteins.

The NLS/NoLS and RBD motifs in several Rev and Rev-like proteins in complex retroviruses are associated with arginine-rich domains [11, 54, 55]. In the present study, the nuclear export activity of the JDV Rev NLS/NoLS mutants targeting the arginine residues was diminished (Mut2, Mut4, Mut6, Mut7 and Mut11) or almost absent (Mut8, Mut10 and Mut12 to Mut16) even though a few protein mutants still localized to the nucleoplasm and/or nucleolus (Fig 4 and S3 Fig). These results suggest that the arginine residues composing the NLS/NoLS are likely part of the RBD that mediates the binding of Rev to the RRE of the viral transcripts, ensuring their transport to the cytoplasm where they are translated. This overlapping of the NLS and the RBD prevents the Rev-viral RNA complexes from going back in the nucleus where the RNA would be spliced [56].

To fulfill its function, the Rev protein must be exported from the nucleus to the cytoplasm. CRM1 (also known as exportin 1) is a member of the karyopherin β family proteins and binds leucine-rich NES for the nuclear export of a protein. HIV-1 and cyclic-AMP-dependent PKI were the first proteins identified harboring a leucine-rich NES that was shown to specifically interact with CRM1. Other cellular and viral proteins with NES were thereafter identified and NES consensus sequences were established and then classified into the HIV-1 Rev class or the PKI class [28]. The present study showed that the JDV Rev protein contains a NES that is CRM1-dependent, as demonstrated by the nuclear export blockage of the protein in presence of LMB (Fig 1). This result is in accordance with the fact that the lentiviral Rev proteins characterized so far are CRM1-dependent for their nuclear export [19, 30, 57]. The Rev sequence deleted in mutant JM6 (aa 113 to 132) was shown to contain a NES as this mutant accumulated in the nucleus of transfected cells regardless of the LMB treatment (Fig 2). Bioinformatics analysis of this region indicated a stretch of 14 residues (^116^M-A-E-L-E-E-R-F-E-D-L-A-L-V^129^) predicted to serve as a NES. When this sequence was analyzed using the Rev(1.4)-EGFP nuclear export assay it was able to recover the NES activity of the HIV-1 Rev protein as determined by the subcellular localization of the protein and the resulting Fn/c ratios (Fig 6). Generating mutants targeting the M, L, F and V hydrophobic residues revealed that ^119^L, ^123^F, ^126^L and ^128^L were key-residues for the NES function of JDV Rev and that the ^129^V was not part of the NES. Even though the ^116^M residue seemed dispensable for the NES function based on the result obtained with mutant M116A in the Rev(1.4)-EFGP nuclear export assay, a decrease in the Rev activity of this mutant, as determined in the CAT reporter assay, was observed (Fig 7). Therefore, this result showed a role for ^116^M, albeit minor, in the NES function. Combined, these results indicated that the NES sequence of JDV Rev is ^116^**M**-A-E-**L**-E-E-R-**F**-E-D-**L**-A-**L**^128^ where the residues associated with nuclear export are indicated in bold type.

The NES of the PKI protein is composed of four key hydrophobic residues with the Φ^1^XXXΦ^2^XXΦ^3^XΦ^4^ consensus sequence where Φ represents hydrophobic residues (Table 2) [40]. It has been shown that a fifth residue (Φ^0^) can enhance the export activity of PKI by reinforcing its interaction with CRM1. Therefore, another consensus sequence (Φ^0^XXΦ^1^XXXΦ^2^XXΦ^3^XΦ^4^) was defined for the PKI NES class [38]. Thus the JDV Rev NES belongs to the latter consensus sequence of the PKI class with the ^116^M, ^119^L, ^123^F, ^126^L and ^128^L residues corresponding to the Φ^0^, Φ^1^, Φ^2^, Φ^3^ and Φ^4^ hydrophobic residues, respectively (Table 2). Consequently, this result obtained for the JDV Rev NES contrasts with the NES consensus of the HIV-1 Rev class. It was also demonstrated that the nature of the Φ residue at any position in a NES sequence of a protein can also impact the strength of its interaction with CRM1 [40]. This was shown by using several substitution mutants targeting the PKI NES such that optimal residues at the Φ positions were identified (Table 3). Accordingly, the JDV Rev NES corresponds to one of the most optimal consensus sequences of the PKI NES. Combined, the results reported here agree with those of a previous report where a NES of the PKI class harboring five hydrophobic aa was described for the BIV Rev protein [14]. However, the BIV Rev NES sequence corresponds to a PKI NES consensus sequence that is less optimal than that of the JDV Rev NES.

**Table 2:** PKI, HIV-1 and JDV Rev NES sequences.

Class consensus sequence	Sequence^1^
PKI class NES	
Former consensus	**Φ**^**1**^ XXX**Φ**^**2**^ XX**Φ**^**3**^X**Φ**^**4**^
PKI WT NES	S N E **L** A L K **L** A G **L** D **I**
New consensus	**Φ**^**0**^XX**Φ**^**1**^ XXX**Φ**^**2**^ XX**Φ**^**3**^X**Φ**^**4**^
Optimal PKI NES	**I** N E **L** A L K **L** A G **L** D **I**
HIV-1 Rev NES	
New consensus	**Φ**^**0**^**Φ**^**1**^ X**Φ**^**2**^ XX**Φ**^**3**^X**Φ**^**4**^
New Rev NES	L Q **L P** P **L** E R **L** T **L**
JDV Rev NES	
Consensus	**Φ**^**0**^XX**Φ**^**1**^ XXX**Φ**^**2**^ XX**Φ**^**3**^X**Φ**^**4**^
Rev NES	**M** A E **L** E E R **F** E D **L** A **L**

^1^The hydrophobic residues composing the NES are shown in bold type (adapted from [40]).

**Table 3:** Hydrophobic residues for PKI NES optimal binding to CRM1.

Position		Residues^1^
**Φ**_**0**_	I	= V = M > L > A = Y > F = W > P
**Φ**_**1**_	L	> I > V = M > F > A > W
**Φ**_**2**_	F	= M > L > I = V > Y > W
**Φ**_**3**_	L	= M > I > V > F > W = A
**Φ**_**4**_	L	> I > M > V > F

^1^ The amino acids composing the most optimal consensus sequence of PKI NES are shown within the box (adapted from [40]).

In conclusion, the results of this study demonstrated that the nucleolar and nuclear localizations of the JDV Rev protein are mediated via NLS and NoLS aa motifs, the latter being novel among Rev and Rev-like proteins. It was also shown that the NES of JDV Rev belongs to the PKI class. It also corresponded to the most optimal consensus sequence of PKI NES and, as such, is novel among lentivirus Rev protein NES. Knowledge of the modes of action and variations among the lentiviral Rev proteins may provide additional targets for drug therapy design against lentiviral infections.

## Supporting information

S1 Fig Subcellular localization of JDV Rev deletion mutant proteins fused to EGFP. Microscopic analysis of Rev deletion mutant proteins fused to EGFP (in green) expressed in MDBK cells 24 h post transfection in absence (-) or presence (+) of leptomycin B (LMB). Cells were fixed and counterstained with DAPI for nucleus visualization (in blue). Images shown are representative of expression pattern observed in 30 cells from three independent experiments (10 cells per experiment). The white bars correspond to a length of 10 μM.
(TIF)

S2 Fig Nuclear export activity of JDV Rev with heterologous lentiviral Rev Response elements (RRE). Nuclear export activity of EGFP-JDV Rev, EGFP-BIV or EGFP-HIV-1 Rev proteins expressed from the appropriate pEGFP-C1 vectors using either the BIV (pDM138) or HIV-1 (pDM128) RRE sequence was determined using a CAT reporter assay. The CAT levels were normalized to the Rev expression as determined by Western blot analysis. Rev activity was determined as the ratio of CAT expression to the basal expression from pDM128 or pDM138 constructs co-transfected with empty pEGFP-C1. The Rev activity mean values ± the standard error about the mean (SEM) were obtained from three independent experiments (triplicate samples per experiment). Significant differences between the EGFP proteins, using a one-way ANOVA followed by a post-hoc Tukey’s multiple-comparison test, are indicated by ** (*P* < 0.005) and *** (*P* < 0.0005).
(TIF)

S3 Fig Subcellular localization of JDV Rev alanine substitution mutant proteins. MDBK cells were transfected with each of the mutant plasmid constructs and incubated for 24 h. Cells were treated with leptomycin B (LMB) for 5 h or left untreated and then fixed, subjected to immunostaining for nucleolin detection (in red) and counterstained with DAPI for nucleus visualization (in blue). Only the results obtained from cells in presence (+) of LMB are shown. The white bars correspond to a length of 10 μM.
(TIF)

S4 Fig Nucleolar localization of the JDV Rev alanine substitution mutant proteins. MDBK cells were transfected with the plasmid constructs encoding either the JDV Rev WT protein or each of the JDV Rev mutant proteins (Mut1 to Mut17), and incubated for 24 h. Cells were left untreated or treated with 5 nM of leptomycin B (LMB) for 5 h and then fixed, subjected to immunostaining for nucleolin detection and counterstained with DAPI for nucleus visualization. CLSM images were obtained at 60x magnification from three independent experiments (10 analyzed cells per experiment). The images were analyzed to determine the Fno/n ratios. Results (mean Fno/n ratio ± the standard error about the mean (SEM), for *n* = 30) are shown for the JDV Rev WT protein and each of the alanine substitution mutant proteins. Significant differences, using an ANOVA followed by a post-hoc Dunnett’s test, between the JDV Rev WT protein and each of the deletion mutants, with and without LMB treatment, are indicated by **** (*P* < 0.00005).
(TIF)
