# Supplementary figures and images for: The Jembrana disease virus Rev protein: Identification of nuclear and novel lentiviral nucleolar localization and nuclear export signals
Source: PLoS One. 2019 Aug 22;14(8):e0221505. doi: 10.1371/journal.pone.0221505 (PMC6706053; doi:10.1371/journal.pone.0221505)

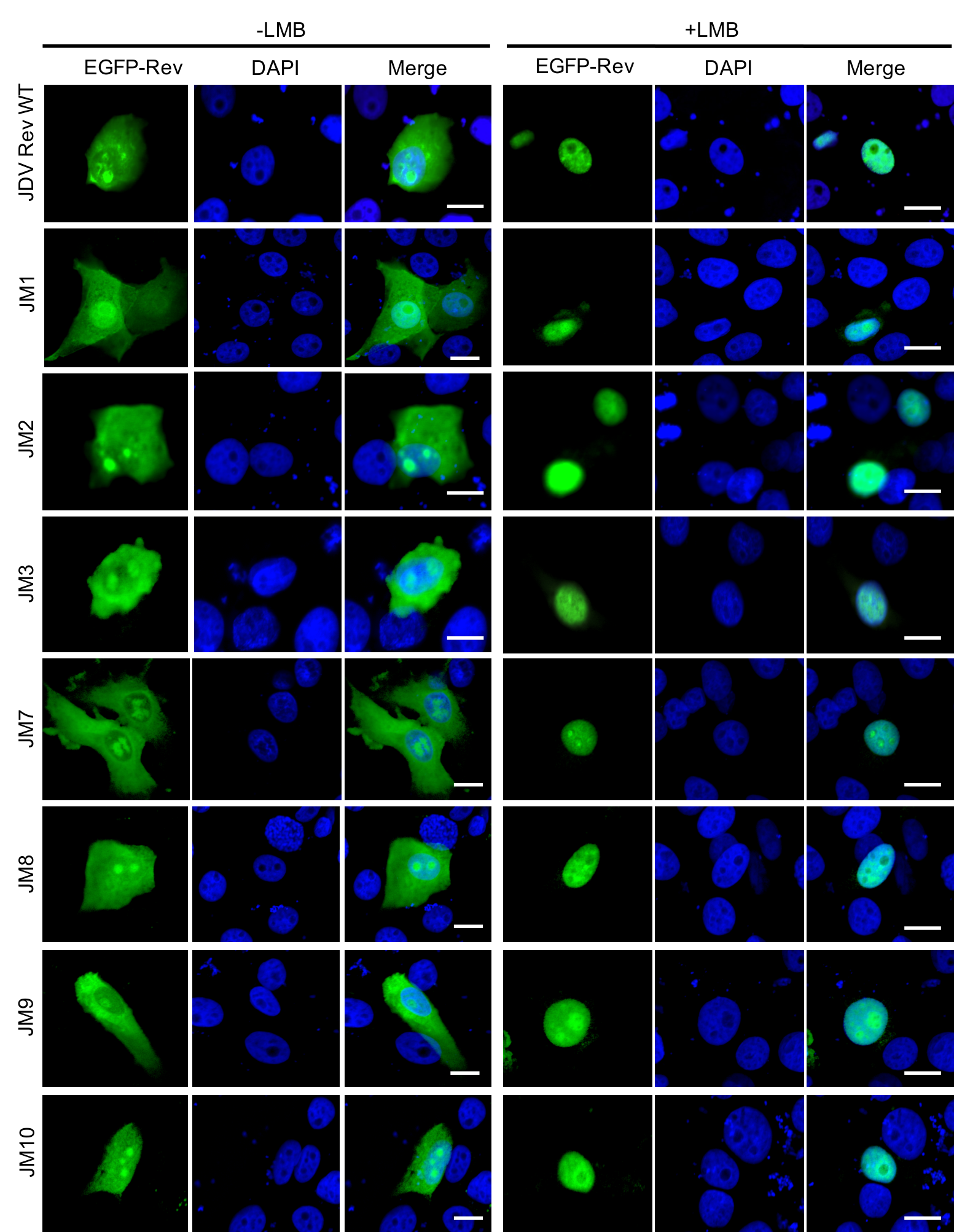

Supplement: S1 Fig — Microscopic analysis of Rev deletion mutant proteins fused to EGFP (in green) expressed in MDBK cells 24 h post transfection in absence (-) or presence (+) of leptomycin B (LMB). Cells were fixed and counterstained with DAPI for nucleus visualization (in blue). Images shown are representative of expression pattern observed in 30 cells from three independent experiments (10 cells per experiment). The white bars correspond to a length of 10 μM. (TIF) [file pone.0221505.s001.tif]

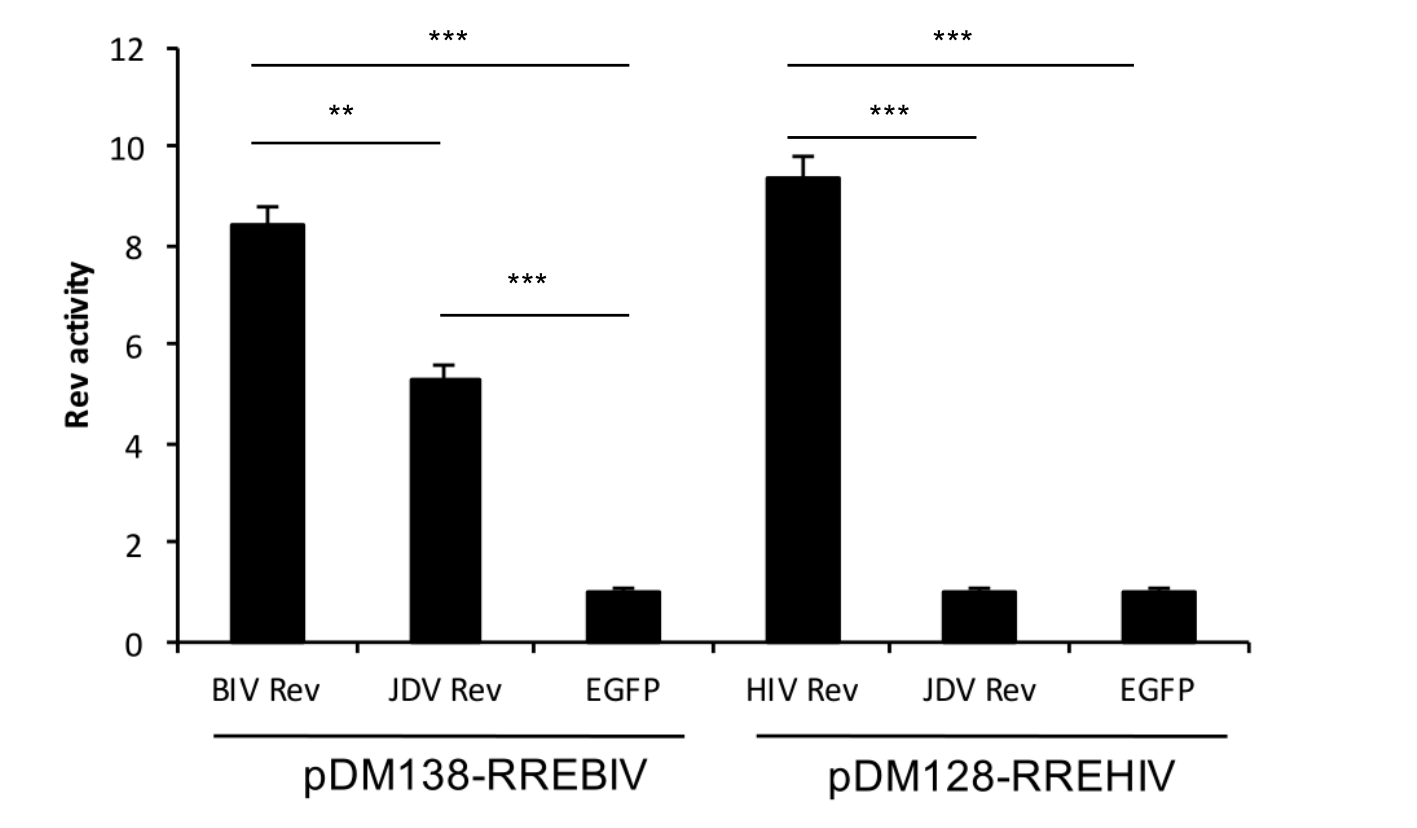

Supplement: S2 Fig — Nuclear export activity of EGFP-JDV Rev, EGFP-BIV or EGFP-HIV-1 Rev proteins expressed from the appropriate pEGFP-C1 vectors using either the BIV (pDM138) or HIV-1 (pDM128) RRE sequence was determined using a CAT reporter assay. The CAT levels were normalized to the Rev expression as determined by Western blot analysis. Rev activity was determined as the ratio of CAT expression to the basal expression from pDM128 or pDM138 constructs co-transfected with empty pEGFP-C1. The Rev activity mean values ± the standard error about the mean (SEM) were obtained from three independent experiments (triplicate samples per experiment). Significant differences between the EGFP proteins, using a one-way ANOVA followed by a post-hoc Tukey’s multiple-comparison test, are indicated by ** (P < 0.005) and *** (P < 0.0005). (TIF) [file pone.0221505.s002.tif]

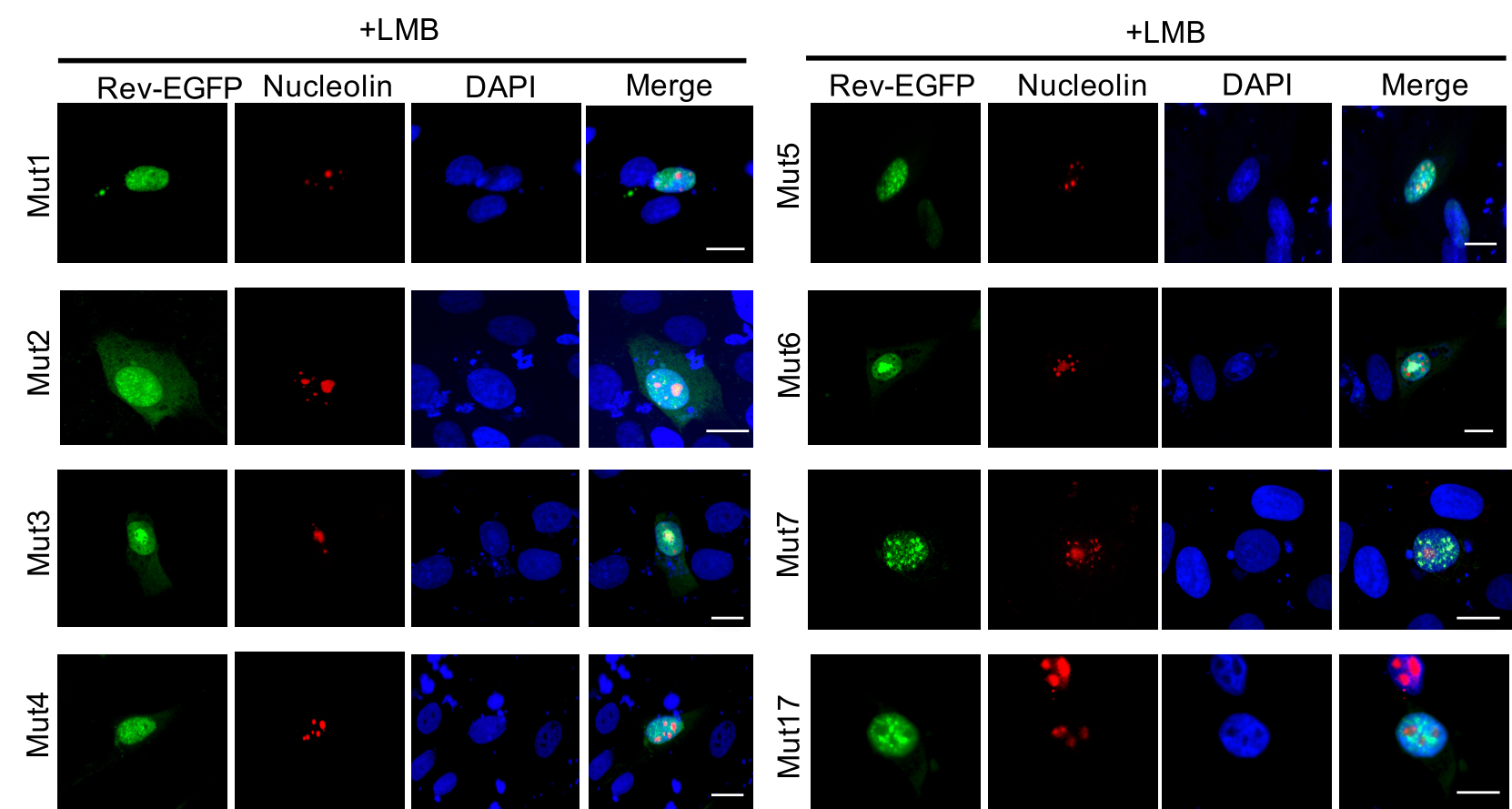

Supplement: S3 Fig — MDBK cells were transfected with each of the mutant plasmid constructs and incubated for 24 h. Cells were treated with leptomycin B (LMB) for 5 h or left untreated and then fixed, subjected to immunostaining for nucleolin detection (in red) and counterstained with DAPI for nucleus visualization (in blue). Only the results obtained from cells in presence (+) of LMB are shown. The white bars correspond to a length of 10 μM. (TIF) [file pone.0221505.s003.tif]

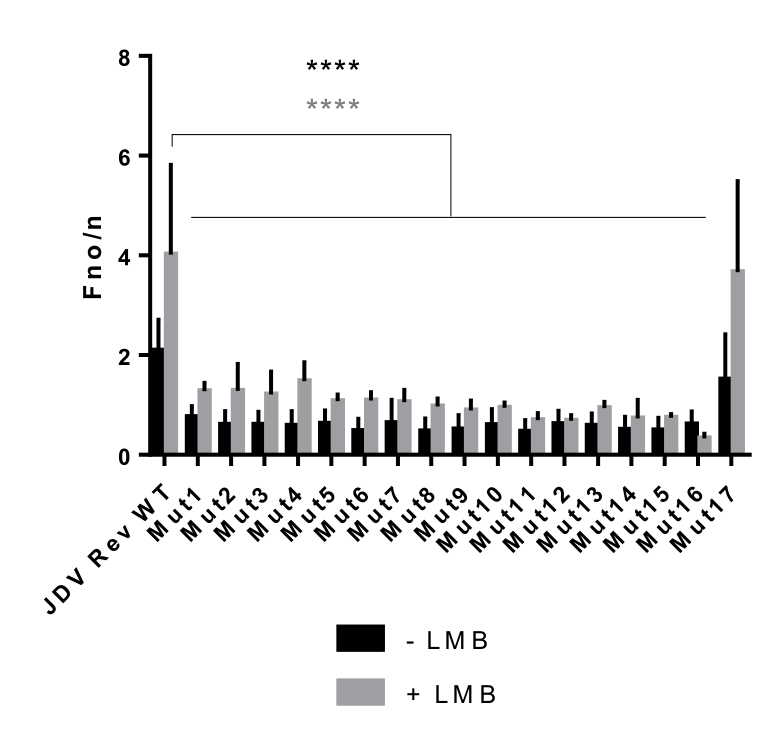

Supplement: S4 Fig — MDBK cells were transfected with the plasmid constructs encoding either the JDV Rev WT protein or each of the JDV Rev mutant proteins (Mut1 to Mut17), and incubated for 24 h. Cells were left untreated or treated with 5 nM of leptomycin B (LMB) for 5 h and then fixed, subjected to immunostaining for nucleolin detection and counterstained with DAPI for nucleus visualization. CLSM images were obtained at 60x magnification from three independent experiments (10 analyzed cells per experiment). The images were analyzed to determine the Fno/n ratios. Results (mean Fno/n ratio ± the standard error about the mean (SEM), for n = 30) are shown for the JDV Rev WT protein and each of the alanine substitution mutant proteins. Significant differences, using an ANOVA followed by a post-hoc Dunnett’s test, between the JDV Rev WT protein and each of the deletion mutants, with and without LMB treatment, are indicated by **** (P < 0.00005). (TIF) [file pone.0221505.s004.tif]
